# Supplementary material for: Surface Microbiome Profiling of Dental Elevators Using Third‐Generation Sequencing: Implications for Infection Control in Dental Practice
Source: Microbiologyopen. 2025 Dec 8;14(6):e70178. doi: 10.1002/mbo3.70178 (PMC12685768; doi:10.1002/mbo3.70178)
Supplement: Supplementary file 1 — customer_backup. [file MBO3-14-e70178-s001.zip › customer_backup/customer_backup/taxa_summary/heatmap/treat/treat.class.reabundance.heatmap.pdf]

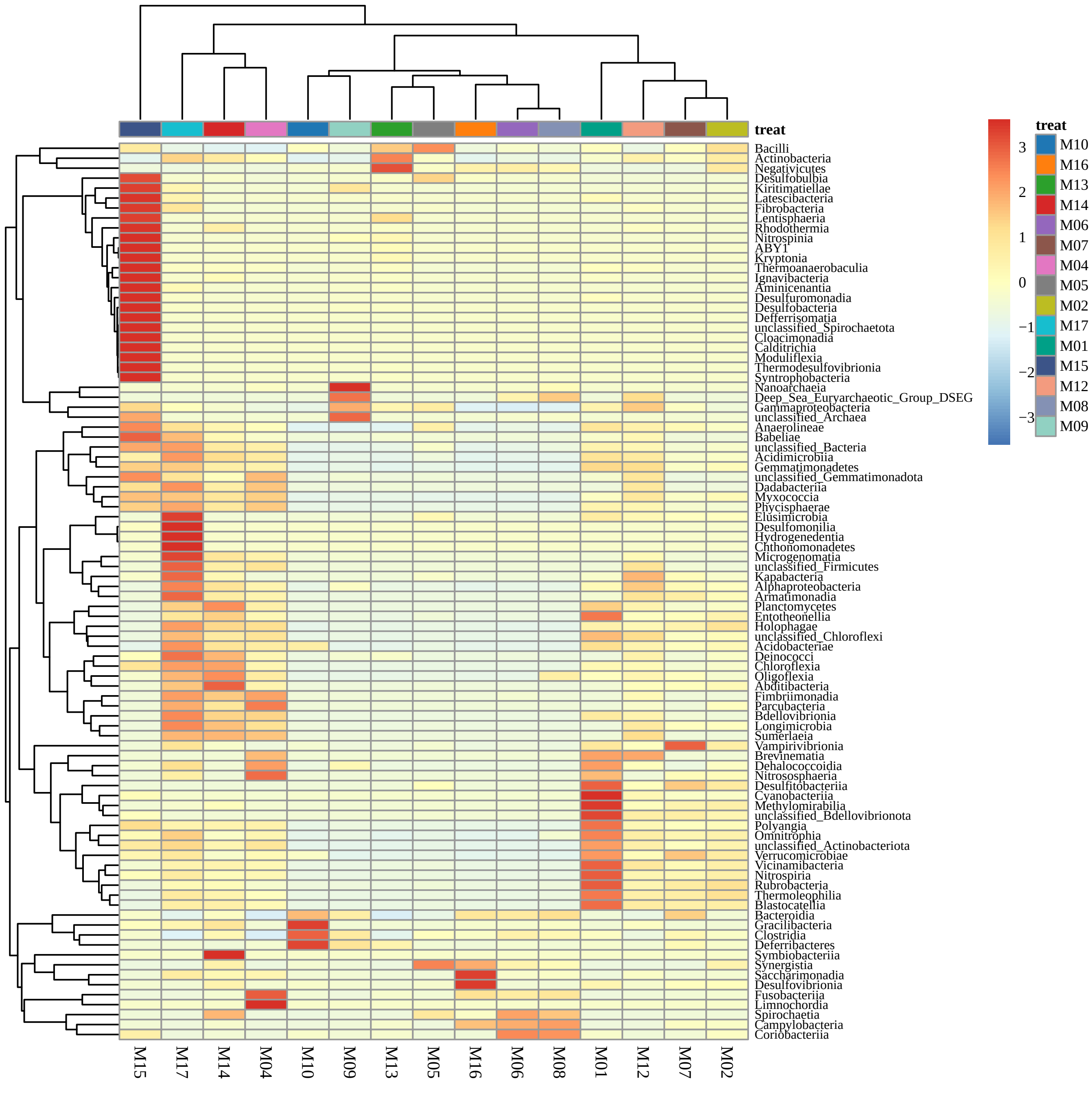

treat
Bacilli
Actinobacteria
Negativicutes
Desulfobulbia
Kiritimatiellae
Latescibacteria
Fibrobacteria
Lentisphaeria
Rhodothermia
Nitrospinia
ABY1
Kryptonia
Thermoanaerobaculia
Ignavibacteria
Aminicenantia
Desulfuromonadia
Desulfobacteria
Defferrisomatia
unclassified_Spirochaetota
Cloacimonadia
Calditrichia
Moduliflexia
Thermodesulfovibrionia
Syntrophobacteria
Nanoarchaeia
Deep_Sea_Euryarchaeotic_Group_DSEG
Gammaproteobacteria
unclassified_Archaea
Anaerolineae
Babeliae
unclassified_Bacteria
Acidimicrobiia
Gemmatimonadetes
unclassified_Gemmatimonadota
Dadabacteriia
Myxococcia
Phycisphaerae
Elusimicrobia
Desulfomonilia
Hydrogenedentia
Chthonomonadetes
Microgenomatia
unclassified_Firmicutes
Kapabacteria
Alphaproteobacteria
Armatimonadia
Planctomycetes
Entotheonellia
Holophagae
unclassified_Chloroflexi
Acidobacteriae
Deinococci
Chloroflexia
Oligoflexia
Abditibacteria
Fimbriimonadia
Parcubacteria
Bdellovibrionia
Longimicrobia
Sumerlaeia
Vampirivibrionia
Brevinematia
Dehalococcoidia
Nitrososphaeria
Desulfitobacteriia
Cyanobacteriia
Methylomirabilia
unclassified_Bdellovibrionota
Polyangia
Omnitrophia
unclassified_Actinobacteriota
Verrucomicrobiae
Vicinamibacteria
Nitrospiria
Rubrobacteria
Thermoleophilia
Blastocatellia
Bacteroidia
Gracilibacteria
Clostridia
Deferribacteres
Symbiobacteriia
Synergistia
Saccharimonadia
Desulfovibrionia
Fusobacteriia
Limnochordia
Spirochaetia
Campylobacteria
Coriobacteriia
M15 M17 M14 M04 M10 M09 M13 M05 M16 M06 M08 M01 M12 M07 M02
3
2
1
0
−1
−2
−3
treat
M10
M16
M13
M14
M06
M07
M04
M05
M02
M17
M01
M15
M12
M08
M09
